# Oxytocin Improves Follicular Reserve in a Cisplatin-Induced Gonadotoxicity Model in Rats

**DOI:** 10.1155/2014/703691

**Published:** 2014-05-15

**Authors:** Oytun Erbaş, Levent Akman, Altuğ Yavaşoğlu, Mustafa Cosan Terek, Tülay Akman, Dilek Taskiran

**Affiliations:** ^1^Department of Physiology, Ege University School of Medicine, 35100 Izmir, Turkey; ^2^Department of Obstetrics and Gynecology, Ege University School of Medicine, 35100 Izmir, Turkey; ^3^Department of Histology and Embryology, Ege University School of Medicine, 35100 Izmir, Turkey; ^4^Division of Medical Oncology, Tepecik Educational and Training Hospital, 35100 Izmir, Turkey

## Abstract

Cisplatin (CP), an antitumor agent, has been shown to cause ovarian injury and dysfunction in both animal and human studies. The present study was conducted to investigate the protective effect of oxytocin (OT) on CP-induced ovarian toxicity in rats. Twenty-one adult female rats were included in the study. Fourteen rats were administered intraperitoneally CP (2 mg/kg/day) twice a week for 5 weeks. Control group (*n* = 7) did not receive any treatment. Following treatment, CP-received rats were randomly divided into two groups and treated with either saline (1 mL/kg/day, *n* = 7) or OT (160 **μ**g/kg/day, *n* = 7) for 5 weeks. Then, ovarian toxicity and effects of OT were evaluated by histomorphological and biochemical analysis. Our findings revealed a significant reduction in the number of follicles at each grade in saline-treated group. AMH level was significantly lower in saline group compared to control (*P* < 0.0005). OT treatment significantly attenuated CP toxicity in ovaries and increased AMH levels compared to saline group (*P* < 0.005). Also, administration of OT lessened lipid peroxidation and prevented glutathione depletion in CP-treated rats (*P* < 0.05). These results indicated that OT could lessen the CP-induced ovarian damage and improve follicular reserve by preventing oxidative damage.

## 1. Introduction

Cytotoxic chemotherapy is used for treating cancers, autoimmune diseases, or hematologic diseases and potentially causes ovarian damage. For women of reproductive age, chemotherapy-induced gonadotoxicity may cause premature ovarian failure resulting from depletion of ovarian reserve [1]. As ovarian function is characterized by cells with rapid turnover, there is a potential similarity to tumor cells in that both are primary targets for chemotherapeutic agents. Ovarian primordial follicular cells have no ability to regenerate, and destruction of these cells leads to ovarian dysfunction, which manifests as premature ovarian failure and infertility [2].

*cis*-Diamminedichloroplatinum (II) (cisplatin, CP) is an antitumor agent effective for treating various human cancers of the breast, brain, head and neck, stomach, lung, bladder, testis, and ovary. Its antitumor activity is attributed mainly to its ability to form platination products in the nuclear DNA [3–5]. However, repetitive treatment with CP is associated with numerous side effects such as nephrotoxicity, neurotoxicity, and reproductive toxicity [3]. In both animal and human studies, CP has been shown to cause ovarian injury, leading to ovarian dysfunction, changes in the estrous cycle, increased follicular apoptosis, and a reduction in the number of anti-Mullerian hormone- (AMH-) secreting follicles [4].

The overproduction of reactive oxygen species (ROS) has been implicated in the pathophysiology of CP-induced cytotoxicity. The increased generation of ROS, mainly O_2_^−^, leads to lipid peroxidation, mitochondrial damage, and DNA injury [6, 7]. Furthermore, ROS may enhance the expression of proinflammatory cytokines through the regulation of p38 mitogen-activated protein kinases (p38MAPK), which could amplify the cytotoxic effects of CP [6, 7].

Oxytocin (OT), a nonapeptide produced in the paraventricular and supraoptic nuclei in the hypothalamus, is essentially associated with uterine contraction during parturition and milk ejection reflex during lactation. OT exerts its central and peripheral effects through G-protein coupled receptors. OT receptors (OTR) have been demonstrated not only in the uterine and myoepithelial tissues, but also in other tissues, including heart, thymus, kidney, pancreas, adipose tissue, and ovaries [8, 9]. Accumulating evidence suggests that OT exerts its cytoprotective effects via antioxidative, antiapoptotic, and anti-inflammatory pathways [10, 11]. Recently, we have shown its beneficial effects against sepsis-induced polyneuropathy, pentylenetetrazol-induced seizures, and rotenone-induced Parkinson's disease model in rats [12–14]. Several observations have indicated the link between OTR stimulation and the activation of phospholipase C (PLC), phospholipase A2 (PLA2), protein kinase C (PKC), and mitogen-activated protein kinase (MAPK) [8, 9, 15, 16].

Although several studies have demonstrated the protective role of OT in various types of tissue injury, there is still lack of data with regard to beneficial effects of this peptide in chemotherapy-induced ovarian gonadotoxicity. Therefore, we conducted the present study to explore whether OT has protective effects on follicle reserve in a CP-induced gonadotoxicity model in rats by evaluating histopathological alterations, plasma AMH levels, and ovary malondialdehyde (MDA) and glutathione (GSH) levels as markers of oxidative stress.

## 2. Materials and Methods

### 2.1. Animals

Twenty-one adult female Sprague-Dawley rats, weighing 220–240 g, were used in the study. Animals were housed in cages and subjected to controlled conditions of temperature (22 ± 2°C) and 12-hour light/dark cycles. They were fed by standard pellet diet and tap water ad libitumalong the study. This protocol was approved by the Institutional Animal Care and Ethical Committee of Ege University.

The estrous stage of each rat was determined by taking a vaginal smear at an interval of 6 to 12 hours. Cells types in the smear were subsequently examined under a microscope according to the staining procedure of Papanicolaou. Only rats with consistent 4-day cycles were included in the study.

### 2.2. Experimental Design

Fourteen rats were administered intraperitoneally cisplatin (2 mg/kg/day; Cisplatin, Koçak Farma) twice a week for 5 weeks. Control group (*n* = 7) did not receive any treatment. Following CP and saline treatment, CP-received rats were randomly divided into two groups and treated with either saline (1 mL/kg/day) or OT (160 *μ*g/kg/day, Pituisan, Ege Vet, Alfasan International B.V., Holland) for 5 weeks. All treatments were performed from 9:00 to 10:00 in the morning. Rats were monitored daily for behavior and health conditions. At the end of treatment period, rats were decapitated and the trunk blood was withdrawn for AMH measurement. Blood samples were centrifuged at 3000 rpm for 10 minutes at room temperature and stored at −30°C until the hormone assay. Both ovaries were obtained from all animals for histological and biochemical evaluation.

### 2.3. Histological Examination

Formalin-preserved ovarian tissues were embedded in paraffin. The ovaries were sectioned at a thickness of 4 *μ*m with a microtome, and the sections were mounted onto glass slides and stained with hematoxylin and eosin. All sections were photographed with Olympus C-5050 digital camera mounted on Olympus BX51 microscope. Morphological analysis of the ovaries was assessed by computerized image analysis system (Image-ProExpress 1.4.5, Media Cybernetics, Inc., USA) on ten microscopic fields per section at a magnification of 40x, by the observer who was blinded to the study groups. Ovarian follicles at different developmental stages were categorized according to standards established by Oktay et al. [17]. Briefly, primordial follicles are localized just below the cortex and contain a single layer of squamous granulosa cells. Primary follicles consist of a single layer of cuboidal granulosa cells. Secondary follicles contain multiple layers of granulosa cells and tertiary follicles are characterized by stratum granulosum and a fluid-filled antral space.

### 2.4. Measurement of AMH Levels

AMH levels were measured in plasma samples using commercially available enzyme-linked immunosorbent assay (Uscn Life Science Inc.) kit. Samples from each animal were determined in duplicate according to the manufacturer's guide. The detection range was 0.156–10 ng/mL.

### 2.5. Measurement of Lipid Peroxides

For biochemical analysis, ovaries were homogenized in ice-cold 150 mM KCl and centrifuged at 5000 ×g for 10 min. The supernatants were used for analysis of lipid peroxides and glutathione levels.

Lipid peroxidation was assessed in each tissue sample by measuring the MDA levels as thiobarbituric acid-reactive substances (TBARS) [18]. Briefly, trichloroacetic acid and TBARS reagent were added to the tissue samples, which were then mixed and incubated at 100°C for 60 min. After cooling on ice, the samples were centrifuged at 3000 rpm for 20 min and the absorbance of the supernatant was read at 535 nm. The MDA levels were calculated from a standard calibration curve using tetraethoxypropane and expressed as nmol/*μ*g protein.

### 2.6. Measurement of Glutathione Levels

GSH content in tissue samples was measured spectrophotometrically according to Ellman's method [19]. In this method, thiols interact with 5,5′-dithiobis-(2-nitrobenzoic acid) (DTNB) and form a colored anion with maximum peak at 412 nm. GSH levels were calculated from the standard calibration curve and expressed as nmol/*μ*g protein.

### 2.7. Measurement of Total Protein Levels

The total protein content in the tissue samples was determined according to Bradford's method using bovine serum albumin as standard [20].

### 2.8. Statistical Analysis

Data analyses were performed using SPSS version 15.0 for Windows. The groups of parametric variables were evaluated by analysis of variance (ANOVA), followed by post hoc Tukey's HSD test. Pearson's correlation analysis was conducted to examine the association between AMH levels and primordial follicle score. Results were given as mean ± standard error of mean (SEM). A value of *P* < 0.05 was accepted as statistically significant.

## 3. Results

### 3.1. Histopathological Examination

**Figure 1:**
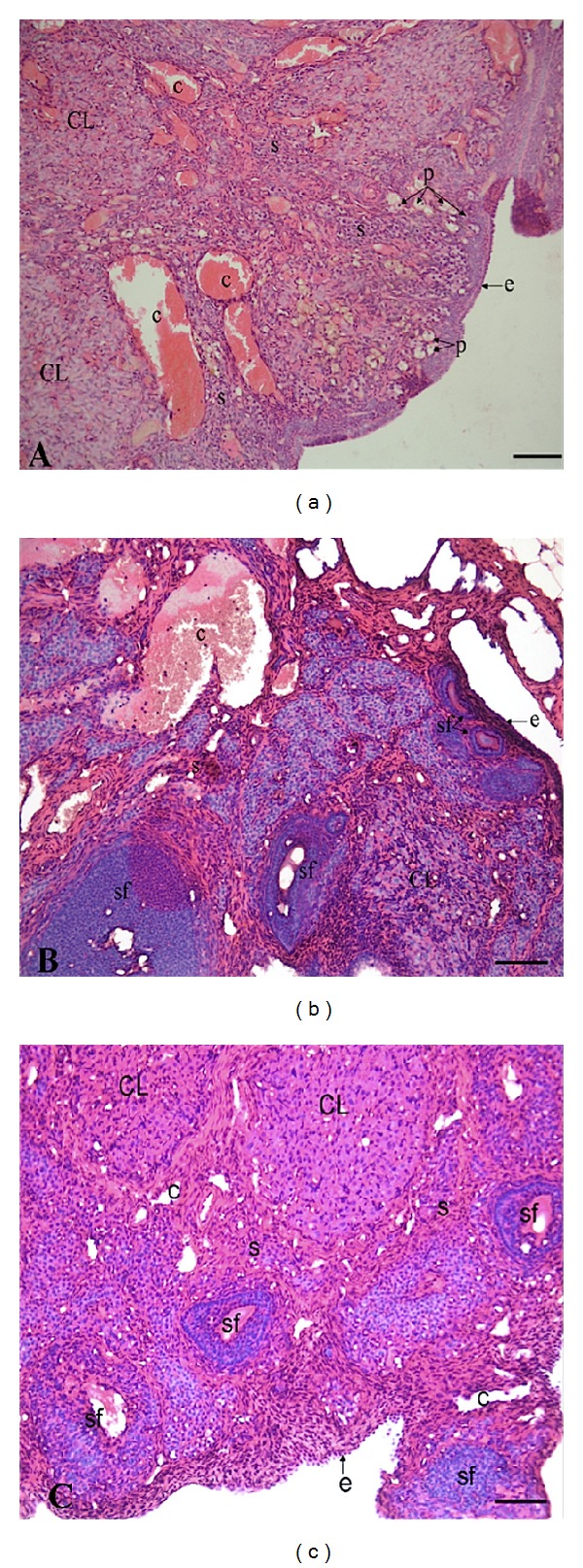
Histological evaluation of ovaries in control (a), CP + saline (b), and CP + OT group (c). Control group showed normal stroma and epithelium whereas cisplatin caused stromal and follicular damage. Treatment of rats with OT significantly ameliorated CP-induced changes in ovaries. Stroma (s), corpus luteum (CL), primordial follicle (p), secondary follicle (sf), surface epithelium (e), and capillary (c). Hematoxylin and eosin staining, ×20 magnification.

Control rats demonstrated normal ovarian morphology, such as regular cuboidal epithelium on the surface, the presence of all types of follicles in the cortex (primordial, primary, secondary, and tertiary), and the presence of capillary vessels in the medullary part of the ovaries (Figure 1(a)). However, CP + saline group revealed a marked reduction in follicle numbers and impaired follicular maturation (Figure 1(b)). Treatment of rats with OT significantly decreased the follicular damage and increased the immature follicles (Figure 1(c)).

### 3.2. Quantitative Analysis of Follicles

**Figure 2:**
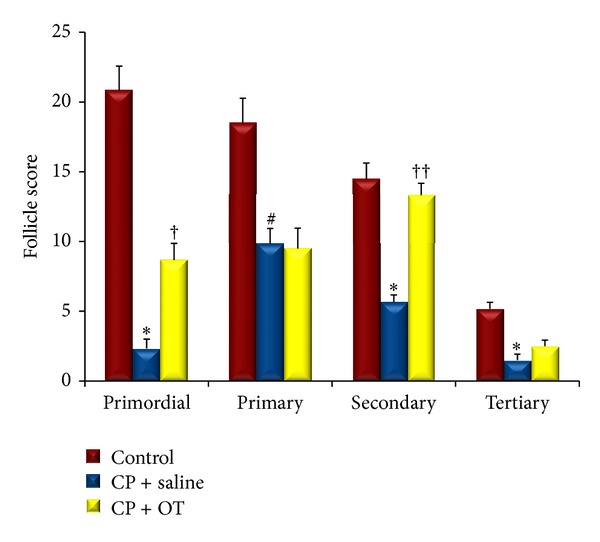
Follicle score in control and study groups. Data were expressed as mean ± standard error of mean (SEM). ∗: different from control (*P* < 0.0005), #: different from control (*P* < 0.05), †: different from saline group (*P* < 0.05), and ††: different from saline group (*P* < 0.0005).

To evaluate the effects of OT on CP-induced ovarian damage, we quantified the primordial, primary, secondary, and tertiary follicles in histological sections. The follicle counts in the control and study groups were shown in Figure 2. ANOVA results revealed significant differences between the groups (*F*_(2,17)_ = 53.93, *P* < 0.0005 for primordial follicles;* F*_(2,17)_ = 12.08, *P* < 0.005 for primary follicles;* F*_(2,17)_ = 31.32, *P* < 0.0005 for secondary follicles; and* F*_(2,17)_ = 18.13, *P* < 0.005 for tertiary follicles). Number of primordial follicles showed a marked reduction in saline-treated group compared to the control group (2.33 ± 0.66 versus 20.83 ± 0.74, resp.; *P* < 0.0005). However, administration of rats with OT significantly enhanced the number of primordial follicles (8.66 ± 1.2, *P* < 0.05). When we compared the primary and secondary follicle counts between the groups, we found a significant decrease in saline-treated group compared to control group (9.83 ± 1.1 versus 18.5 ± 1.76 and 5.66 ± 0.49 versus 14.5 ± 1.11; *P* < 0.05 and *P* < 0.0005, resp.). Treatment of rats with OT successfully prevented the CP-induced damage in secondary follicles (13.33 ± 0.84, *P* < 0.0005), whereas no significant change was observed in the number of primary follicles. In addition, tertiary follicle count was significantly reduced in saline-treated group when compared with control group (1.5 ± 0.42 versus 5.16 ± 0.47, resp.; *P* < 0.0005). However, the influence of OT administration on follicle number was not statistically significant (2.5 ± 0.42; *P* = 0.28).

### 3.3. Biochemical Evaluation

**Figure 3:**
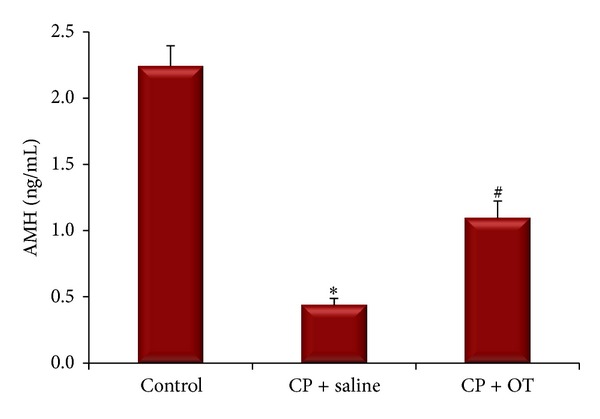
Alterations in AMH levels of both saline- and OT-treated rats in CP-induced ovarian toxicity. ∗: different from control (*P* < 0.0005) and #: different from saline group (*P* < 0.005). Data were expressed as mean ± standard error of mean (SEM).

Circulating AMH levels were measured to determine the changes in the follicle pool of CP-treated rats.  Figure 3 depicts the alterations in AMH levels of the study groups. ANOVA results revealed significant differences between the groups (*F*_(2,18)_ = 47.81, *P* < 0.0005). Compared to the control group, plasma AMH levels were significantly reduced in saline-treated group (2.24 ± 0.15 ng/mL versus 0.44 ± 0.05 ng/mL, resp.; *P* < 0.0005). The treatment of the rats with OT for 5 weeks significantly increased the AMH levels compared with saline-treated group (1.11 ± 0.16 ng/mL, *P* < 0.005).

**Figure 4:**
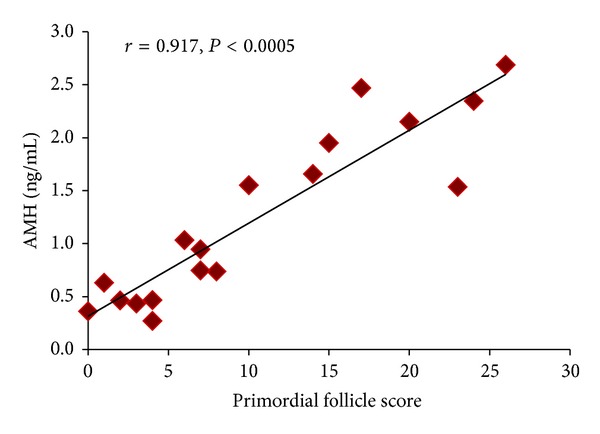
Relationship between the primordial follicle score and AMH level. A significant negative correlation was observed between the primordial follicle scores and AMH levels (*r* = 0.917, *P* < 0.0005).

We also analyzed the relationship between AMH levels and primordial follicle score (Figure 4). Correlation analysis showed that the plasma AMH levels correlated strongly with the numbers of primordial follicles (*r* = 0.917, *P* < 0.0005).

**Figure 5:**
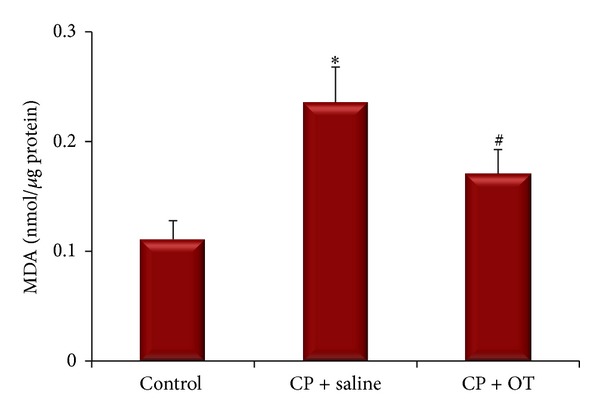
Effect of oxytocin treatment on ovary MDA levels. ∗: different from control (*P* < 0.001) and #: different from saline group (*P* < 0.05). Data were expressed as mean ± standard error of mean (SEM).

**Figure 6:**
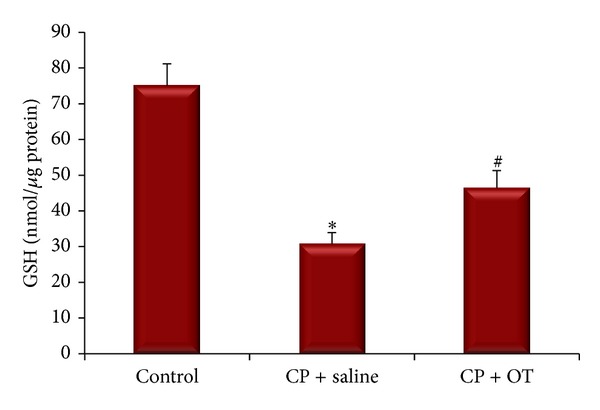
Effect of oxytocin treatment on ovary GSH levels. ∗: different from control (*P* < 0.005) and #: different from saline group (*P* < 0.05). Data were expressed as mean ± standard error of mean (SEM).

The ovary MDA and GSH contents were measured to assess the effects of OT treatment on the oxidative and antioxidative status in study groups. Figures 5 and 6 represent the alterations in MDA and GSH levels of the study groups. ANOVA results revealed significant differences between the groups (*F*_(2,18)_ = 3.68, *P* < 0.005 for MDA and* F*_(2,18)_ = 4.28, *P* < 0.0005 for GSH). Post hoc Tukey's HSD test demonstrated a significant difference in ovarian MDA content in the saline group when compared with the control group (0.24 ± 0.03 versus 0.11 ± 0.01 nmol/*μ*g protein, *P* < 0.001). However, treatment of the rats with OT significantly decreased MDA levels (0.17 ± 0.02 nmol/*μ*g protein, *P* < 0.05). Tissue GSH level in the saline-treated group was significantly lower than in the control group (30.77 ± 3.14 versus 75 ± 6.22 nmol/*μ*g protein, *P* < 0.005). Administration of OT significantly prevented the decrease in ovary GSH content when compared to saline treatment (46.42 ± 4.87 nmol/*μ*g protein, *P* < 0.05).

## 4. Discussion

Women may undergo premature ovarian failure and amenorrhea because of gonadotoxic side effects of chemotherapeutic agents. As indicated in previous reports, there is a direct effect of cytotoxic agents on primordial follicles [21, 22]. Perez et al. reported that mice exposed to chemotherapeutic agents showed apoptosis of granulosa cells in primordial follicles [21]. Meirow examined human ovarian slices following exposure to cisplatin in vitro and found histological evidence of apoptosis in the granulosa cells and destruction of primordial follicles [22].

Recently, the protective effects of drugs on chemotherapy-induced ovarian dysfunction are usually evaluated by the measurement of follicle-stimulating hormone (FSH), estradiol, inhibin B, and AMH in plasma samples [23, 24]. AMH, also known as Mullerian inhibiting substance (MIS), is a dimeric ovarian protein produced by the small growing follicles and is responsible for numerous reproductive functions in female. AMH appears to be a more sensitive marker of ovarian reserve than the other hormones, because circulating AMH levels decrease with aging and reflect fertility problems in women [23–27]. AMH has been recognized as one of the ovarian growth factors that control the rate at which primordial follicles are recruited for further growth. In addition, because AMH level is strongly correlated with the size of the follicle pool and because of the lack of cycle variations, plasma levels of AMH are a good candidate for assessment of other ovarian dysfunctions.

According to the results of the present study, histopathological assessment of follicles revealed the impaired integrity of the surrounding granulosa layer in most of the follicles examined and significantly reduced number of primordial cells in CP-treated rats. Also, AMH level was markedly lower in the plasma samples of CP-treated rats compared to control rats. The damaging effects of CP on ovarian follicles have been previously reported by numerous investigators [4, 5, 28]. For instance, Yucebilgin et al. observed that single dose of CP (5 mg/kg) and paclitaxel (7.5 mg/kg) could significantly damage primordial follicles in rats [4]. Similarly, Ozcelik et al. demonstrated that the administration of paclitaxel and cisplatin chemotherapy alone or in combination had a cytotoxic effect on follicles at all stages of follicle development [28]. Recently, it has been reported that increasing doses of CP cause a dose-dependent decrease in the baseline ovarian expression of AMH and serum AMH levels in rats [5].

Previous studies have suggested that OT could exert antioxidant properties and modulate immune and anti-inflammatory responses in several inflammation and sepsis-induced animal models [10–13]. Senturk et al. have indicated the potential protective effect of OT in the urinary bladder tissue against ischemia/reperfusion injury in rats [29]. In a recent study, Rashed et al. have showed that OT could successfully reduce the monocellular infiltration and tubular injury in CP-induced nephrotoxicity by decreasing the gene expression of NADPH oxidase and P38MAPK, nitric oxide, myeloperoxidase, and lipid peroxidation and also increasing tissue levels of antioxidants such as GSH and superoxide dismutase [7].

MDA, an aldehyde product of lipid peroxidation, is commonly used as a marker of oxidative stress in cells. MDA may modify membrane permeability and fluidity by interrupting ionic transport and enzymatic activity in the cell [6, 7, 30]. Similarly, tissue GSH level is an indicator of antioxidant status in multiple systems. The thiol group in its cysteine moiety provides antioxidant properties, which inhibits harmful effects of ROS on cellular components [6, 7, 30]. In the present study, we found a significant increase in ovary MDA levels in saline-treated group whereas OT effectively lessened lipid peroxidation due to CP toxicity. Also, our findings suggested that OT might have useful effects on ovarian GSH depletion in CP-induced cytotoxicity.

It is evident that the great diversity of suggested roles of OT is paralleled by a diversity of its signaling pathways within the cell. Although there is only one type of OTR, its activation can generate various cell functions. In dependence on G-protein coupling, OTR can give rise to proliferative or inhibitory effects [8, 9, 31]. Indeed, several reports have indicated that OT could activate complex intracellular networks and different kinases using different intermediates. For instance, phosphorylation of extracellular signal-regulated kinase 2 (ERK 1/2) and Akt may contribute to the cytoprotective and proliferative effects of OT [32]. In a rabbit model of myocardial ischemia-reperfusion injury, Kobayashi et al. revealed that OT could improve cardiac functions via triggering cell-survival signals, such as pAkt, pSTAT3, pERK (extracellular signal-regulated kinase), and Bcl-2 [33].

In the present study, treatment of rats with 160 *μ*g/kg/day OT for 5 weeks successfully restored the CP-induced histopathological changes and improved follicle pool in rats. To our knowledge, there is no use of oxytocin in a similar protocol and therefore we cannot compare the dosage regime used in the present study with others. However, OT has been found to produce anti-inflammatory, antioxidant, and cytoprotective effects in the dosage range of 100 *μ*g/kg to 1000 *μ*g/kg in several experimental models in rats [10, 12–14].

In conclusion, the findings of the present study indicate that pharmacological doses of OT could attenuate the deleterious effects of cisplatin on ovary and improve ovarian reserve in rats. On the basis of these data, we suggest that OT supplementation may offer a new therapeutic strategy against chemotherapy-induced ovarian toxicity. However, additional experimental and clinical studies are required to test whether the use of OT affects therapeutic efficacy of cisplatin in gynecological cancers.
